# Efficacy of using a 3D printed lumbosacral spine phantom in improving trainee proficiency and confidence in CT-guided spine procedures

**DOI:** 10.1186/s41205-018-0031-x

**Published:** 2018-10-10

**Authors:** Yi Li, Zhixi Li, Simon Ammanuel, Derrick Gillan, Vinil Shah

**Affiliations:** 1 Department of Radiology and Biomedical Imaging, University of California, San Francisco, 505 Parnassus Avenue, M-391, San Francisco, CA 94143-0628 USA; 2 School of Medicine, University of California, San Francisco, 505 Parnassus Avenue, San Francisco, CA 94143 USA; 3 Lucile Packard Children’s Hospital, Stanford University, 725 Welch Road, Palo Alto, CA 94304 USA

**Keywords:** 3D printing, Training model, Spondylosis, Spine, Spine procedure, Nerve, Facet

## Abstract

**Background:**

Minimally-invasive spine procedures provide targeted, individualized diagnosis and pain management for patients. Competence in these procedures is acquired through experience and training. We created a 3D printed model of a degenerative lumbosacral spine with scoliosis and spondylosis, using materials that mimic bone and soft tissue density under CT. In this study, we evaluate the efficacy of using such a spine model to improve novice trainee confidence and proficiency in performing CT-guided facet joint injections.

**Results:**

Thirteen medical students with no prior exposure to CT-guided spine procedures were divided into two groups. Both groups received an introductory didactic lecture, as well as identical pre- and post- test assessments. The Training group (7 students) received two separate training sessions using the simulation model. The Control group (6 students) received only one training session. The Training group demonstrated significantly fewer needle readjustments during the second simulation session, compared with the first session (*p* = 0.005). Both groups demonstrated significant increase in confidence in ability to perform CT-guided spine procedures on the post-test (*p* = 0.004 for the Control group and *p* = 0.00001 for the Training group).

**Conclusion:**

A 3D printed lumbosacral spine phantom with realistic spondylosis can be made to facilitate novice training in minimally-invasive spine procedures. Training using a realistic lumbosacral spine model helps novices acquire the skills and confidence to perform CT-guided spine procedures.

## Background

Minimally-invasive computed tomography (CT)- or fluoroscopically-guided spine procedures are commonly performed in radiology departments to provide targeted, individualized diagnosis and pain management for patients [1, 2]. Imaging guidance with CT or fluoroscopy allows for direct visualization of needle trajectory and eventual needle endpoint.

Competence in these procedures is acquired through experience and training. Novice trainees usually learn to perform such procedures through real-time interactions with patients, under the supervision of an experienced operator. Learning such procedures on real patients may subject patients to a greater than necessary number of needle readjustments in order to obtain the proper needle positioning, ultimately resulting in a longer procedure and a higher radiation dose. Additionally, errors in proficiency made by trainees who have not yet developed technical expertise in these procedures may lead to unnecessary patient risk.

Despite the increased performance of these imaging-guided minimally-invasive spine procedures over the past decade, few studies to date have described effective methods of teaching such procedures to novice operators [3–5]. Several prior studies have described the creation and usage of phantom models in the training of imaging-guided needle-based procedures and surgical procedures, such as minimally invasive arthroscopy [4–10]. The use of a phantom spine model for training CT-guided spine procedures would reduce patient risk and radiation exposure during the training process. Commercially-available spine phantom models often lack the degenerative anatomic details that make real-life procedures challenging. Based on these factors, we created a 3D printed model of a degenerative lumbosacral spine with scoliosis and spondylosis, using materials that mimic bone and soft tissue density under CT. In this study, we evaluate the efficacy of using such a spine model to improve novice trainee confidence and proficiency in performing CT-guided facet joint injections.

## Methods

### Participants

This study was granted exemption by the Institutional Review Board of the University of California, San Francisco. Thirteen first and second year medical student participants with no prior experience in CT-guided minimally-invasive spine procedures were randomly assigned to two groups. One group was designated the “Training group,” and was scheduled to receive two procedural training sessions using the lumbar spine phantom. The second group was designated the “Control group,” and was scheduled to receive only one training session. Based on variability in student scheduling and availability, the Training group comprised of 7 students and Control group comprised of 6 students.

### Phantom

**Fig. 1:**
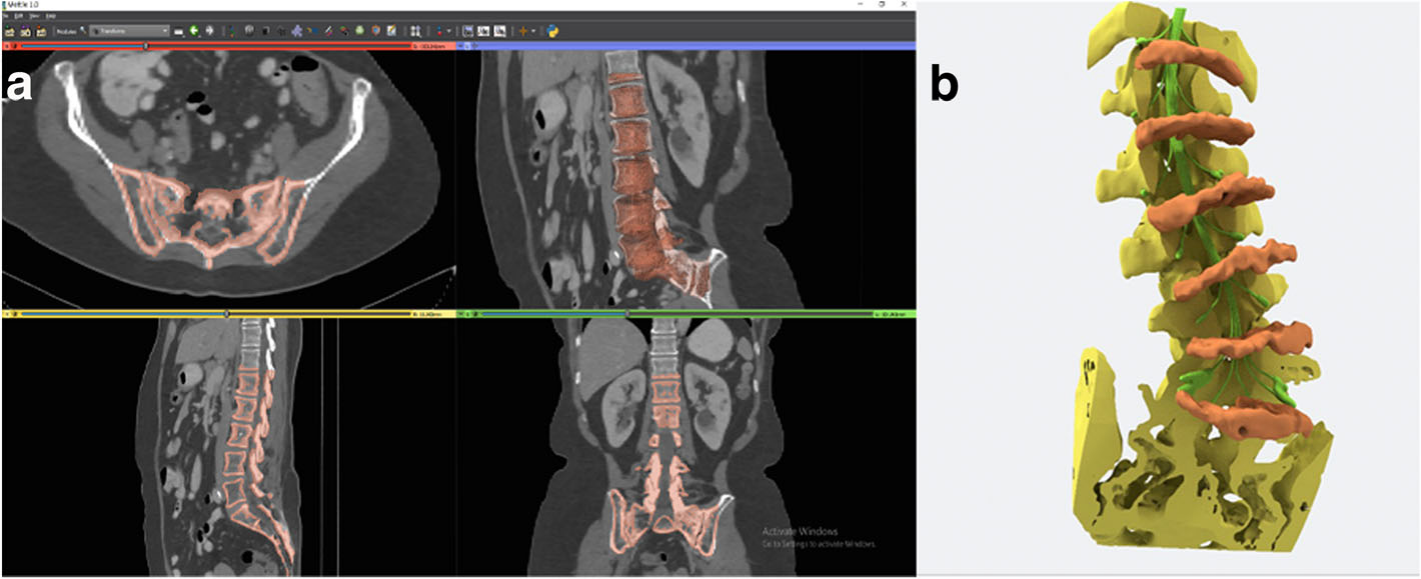
**a** Screenshot of lumbar spine segmentation in four planes. **b** Computer model of segmented degenerative lumbar spine with vertebral bodies removed, demonstrating segmentation of the degenerative intervertebral discs and nerve roots

Images from a diagnostic-quality CT of the lumbosacral spine were used to reconstruct the osseous and soft tissue anatomy for 3D printing. The CT was obtained without intravenous contrast with the following parameters: kVp 140, mA 192, 1.25 mm slice thickness, and reconstructed using bone kernel algorithm. The lumbar spine demonstrated levoconvex scoliosis, significant loss of intervertebral disc height, endplate osteophytosis and facet arthropathy. 3D Slicer was used for initial 3D segmentation (Fig. 1), using both automated and manual segmentation techniques to ensure preservation of fine osseous detail. Materialise Mimics software version 19.0 (Materalise, Leuven, Belgium) was subsequently used for touch up of fine osseous detail, such as degeneration in the facet joints. The osseous model was exported as a stereolithography (STL) file. Separately, the intervertebral discs and nerve roots, including the dorsal root ganglia, were manually outlined and drawn using Materialise Mimics and 3-matic software (Materalise, Leuven, Belgium).

The STL file was printed in several steps. The osseous structures were printed using calcium sulfate hemihydrate, with minimum detail of 0.8 mm, using a 3D Systems Projet 660pro commercial printer (3D Systems, Rock Hill, South Carolina). The nerve roots and discs were both printed using Stratasys Polyjet FLX980 Tango Black. Subsequently, the osseous and non-osseous components of the spine were assembled together. The model was then embedded in a semi-transparent gelatin mold to simulate soft tissue consistency during the needle procedure. This gelatin mold could be melted and remolded between sessions, to eliminate the presence of permanent needle tracks and skin entry sites. During the student practice sessions, a thin coat of oil-based paint was painted over the intended skin surface of the gelatin, masking its transparency. Subsequently, the lumbosacral spine model was imaged under CT for confirmation of differential density of the various structures.

### Knowledge-based examination and surgical simulation

All participants initially underwent a pre-test knowledge-based examination, which included three multiple-choice questions regarding the most common source of low back pain in the adult population and to identify on axial CT images a nerve root and a facet joint. One additional question asked participants to rate their confidence in performing CT-guided spine procedures on a scale of 1–10.

Next, each group was given a 15 min introductory didactic lecture, which provided an introduction to the epidemiology and causes of adult low back pain, spinal anatomy including common areas of degeneration and the role of inflammation in the genesis of low back pain and radiculopathy, the appearance of such spinal anatomy on axial CT, and a technical introduction to the performance of facet joint injections.

Subsequently, each group was taken to the CT scanner, where all participants practiced CT-guided facet injections on the lumbar spine phantom for approximately 3 h, under the guidance of experienced instructors (VS and YL with combined 6 years of spine procedural experience). To increase overall efficiency during the training sessions, several students simultaneously practiced facet injections at different vertebral body levels. The number of re-adjustments required to manipulate the needles into the facet joints was recorded for each participant. Because multiple participants simultaneously performed facet injections, individual radiation dose and individual performance time were not tracked during the training sessions. After each round of needle readjustments, all vertebral body levels were imaged, rather than the individual participant’s level, so radiation dose would have reflected all levels. Similarly, the overall needle adjustment time would reflect the amount of time it took all participants to finish adjustments, not the participant’s individual time. Thus, the number of needle re-adjustments per participant was used as a surrogate for both radiation dose and time, under the assumption that more needle readjustments require more scans, thus leading to higher radiation dose and longer overall procedure time.

The training group was subsequently brought back two weeks later for a repeat 3 h interventional session, and the number of needle readjustments required to achieve final needle position in the facet joint was again recorded for each participant.

A 64slice CT scanner (GE VCT 64D, General Electric Healthcare, Milwaukee, Wisconsin) was used to perform all CT-guided procedures. Images were acquired and immediately viewed on the console to plan the trajectory for subsequent needle advancement. 6 cm 22 g spinal needles were used to perform all needle procedures.

At the end of the last training session for both groups, a post-test knowledge-based examination, composed of identical questions as the pre-test, was administered.

### Statistical analysis

Student t-test (two-tailed, alpha = 0.05) was used to compare inter-group and pre- and post-training differences in several categories: written exam performance; confidence in ability to perform spine procedures; and number of readjustments needed to achieve final needle position during practice facet blocks using the phantom model.

## Results

### Phantom model

**Fig. 2:**
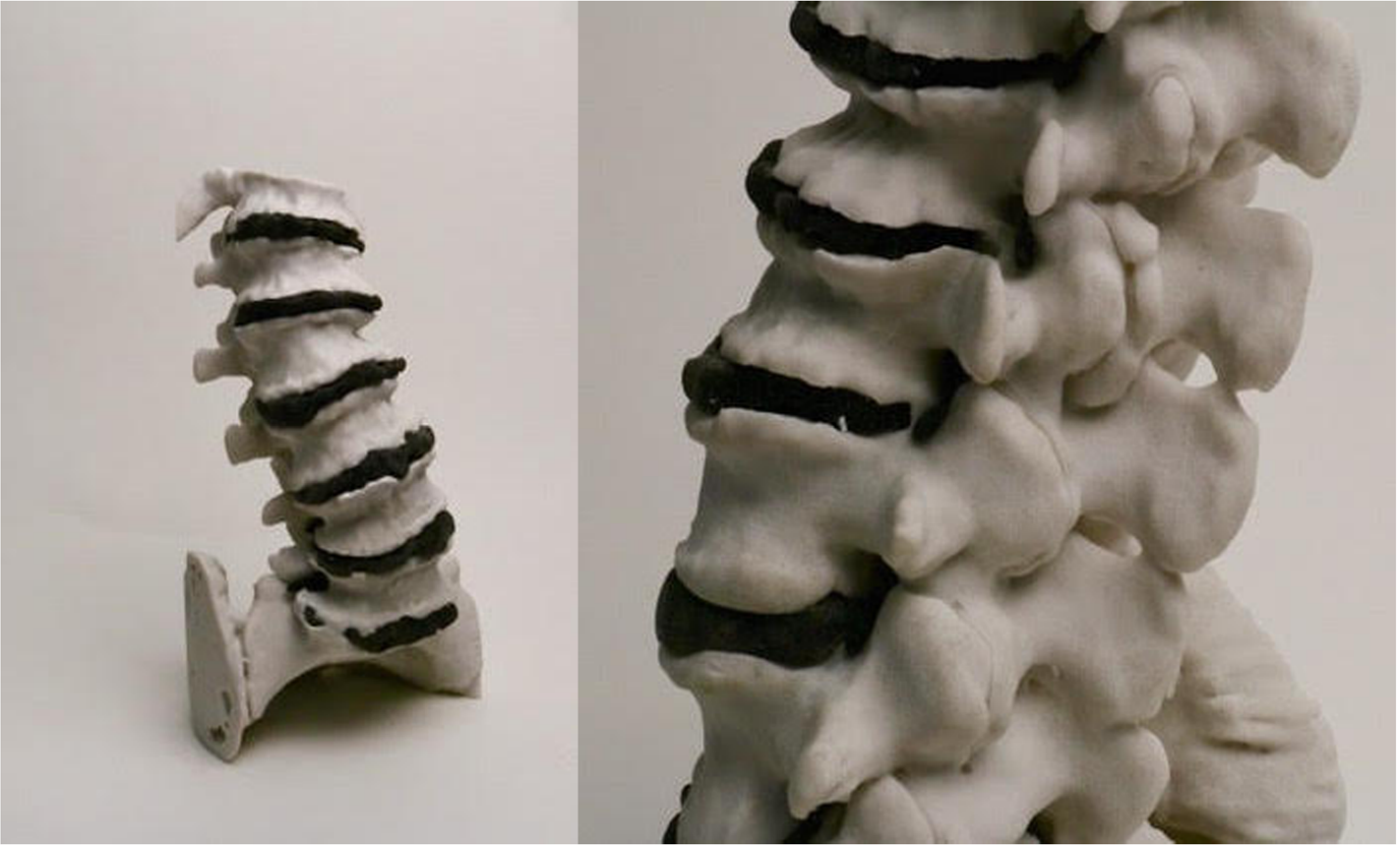
3D printed model of a lumbosacral spine with realistic spondylosis, including levoconvex scoliosis and degenerative facet arthropathy, modeled from a real patient CT. Ivory-colored vertebral bodies were printed using calcium sulfate hemihydrate. Black-colored intervertebral discs were printed using Stratasys Polyjet FLX980 Tango Blacrk

**Fig. 3:**
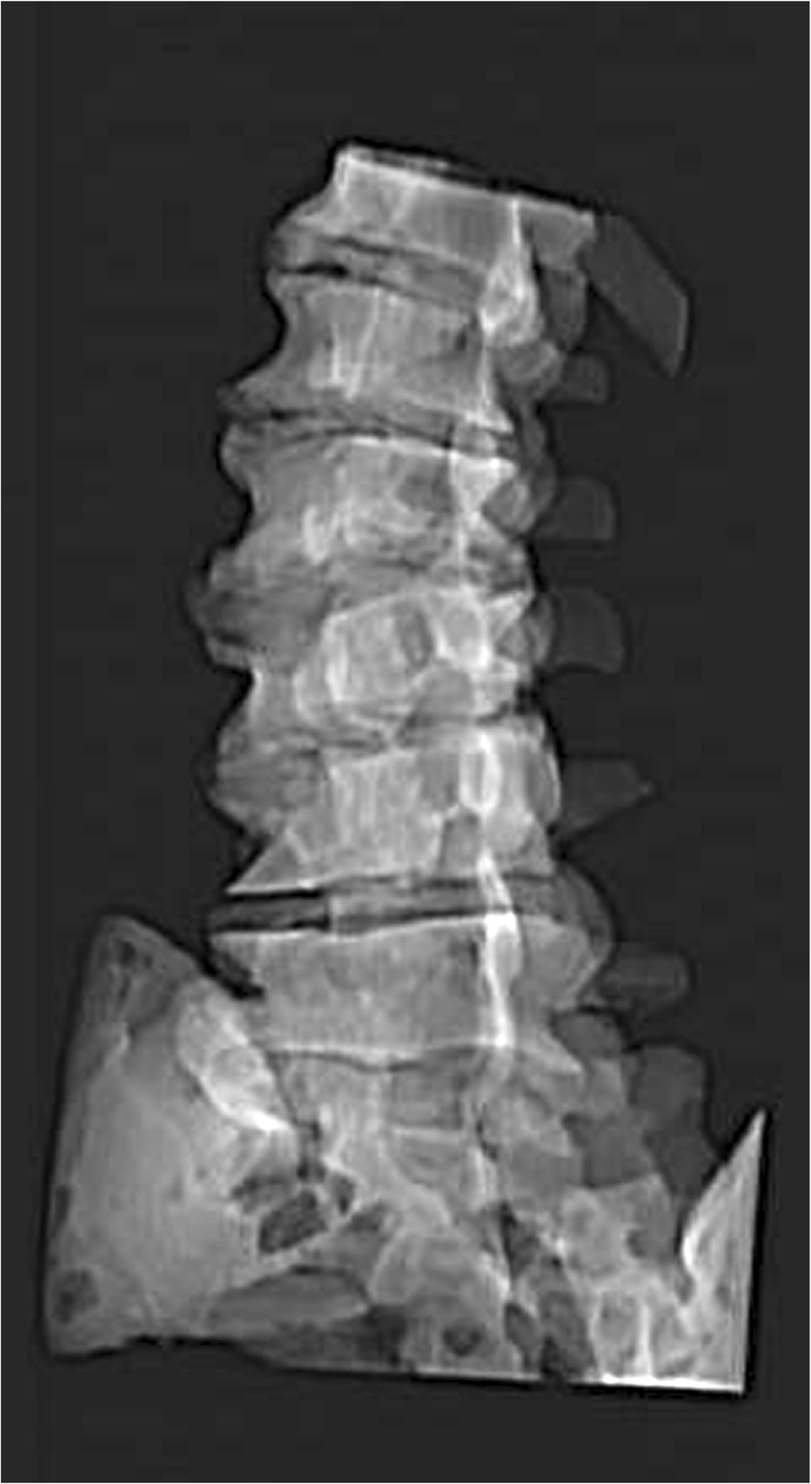
The osseous structures of the model are visible under fluoroscopy

**Fig. 4:**
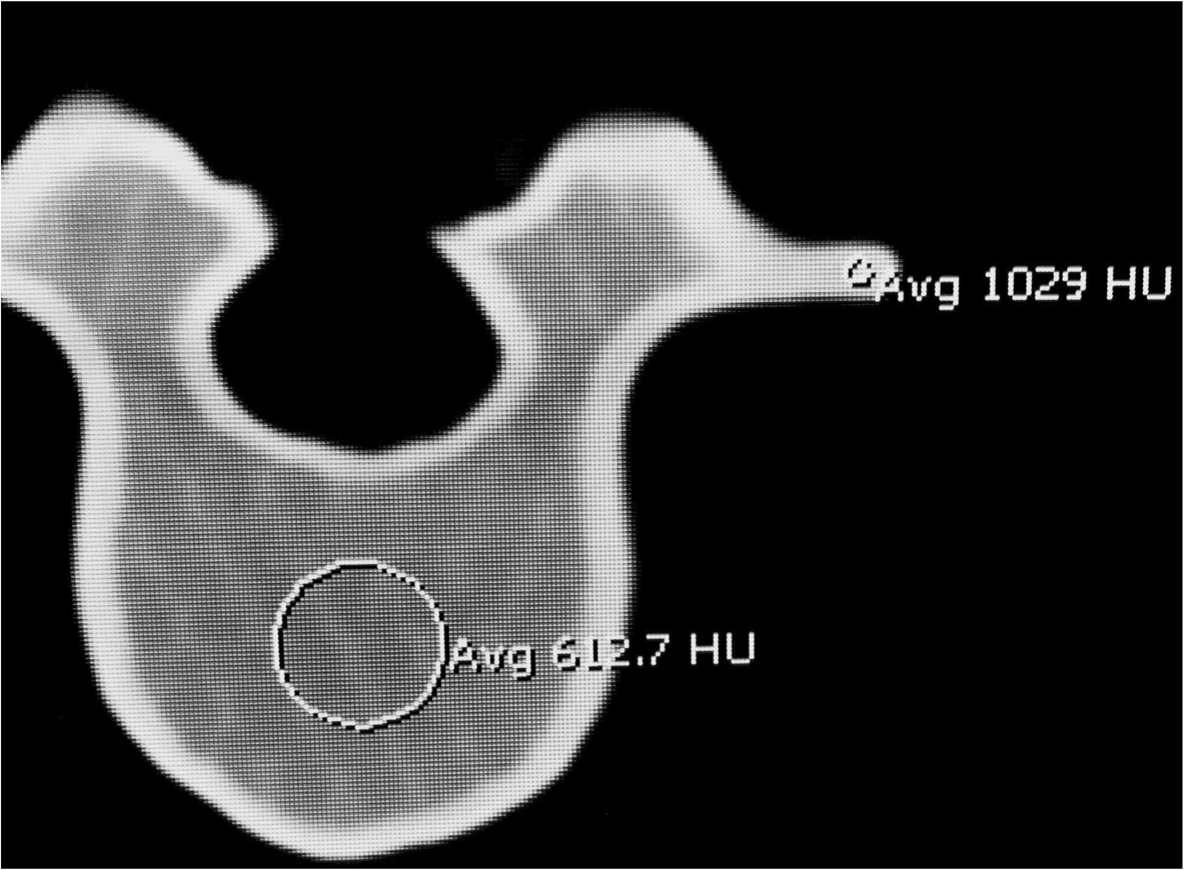
Under CT, the lumbar spine model demonstrates Hounsfield Units that mimic cortical and medullary bone

**Fig. 5:**
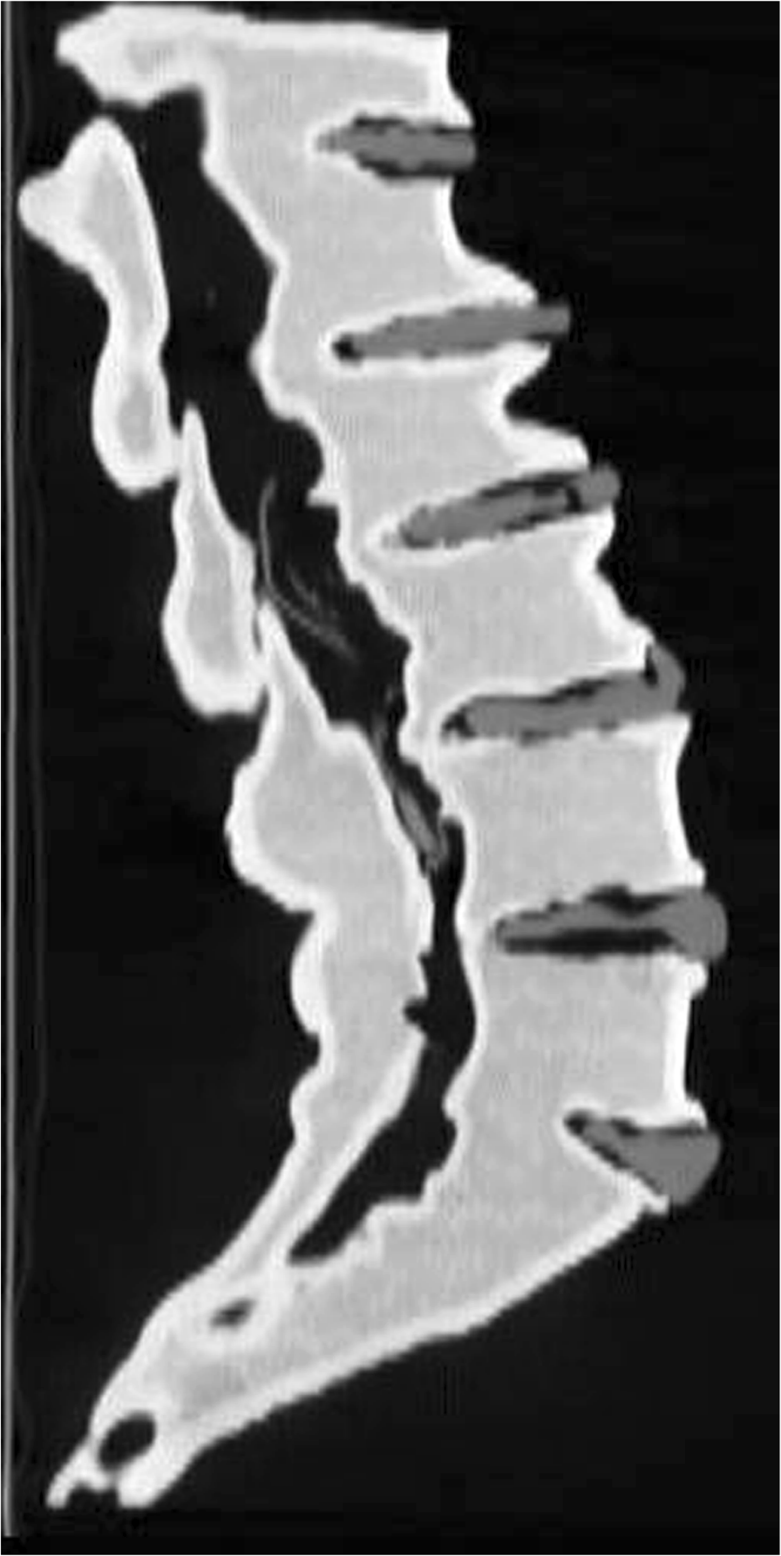
Sagittal reformat of CT of the lumbar spine model, demonstrating differential densities and ability to distinguish the vertebral bodies, discs and nerve roots

**Fig. 6:**
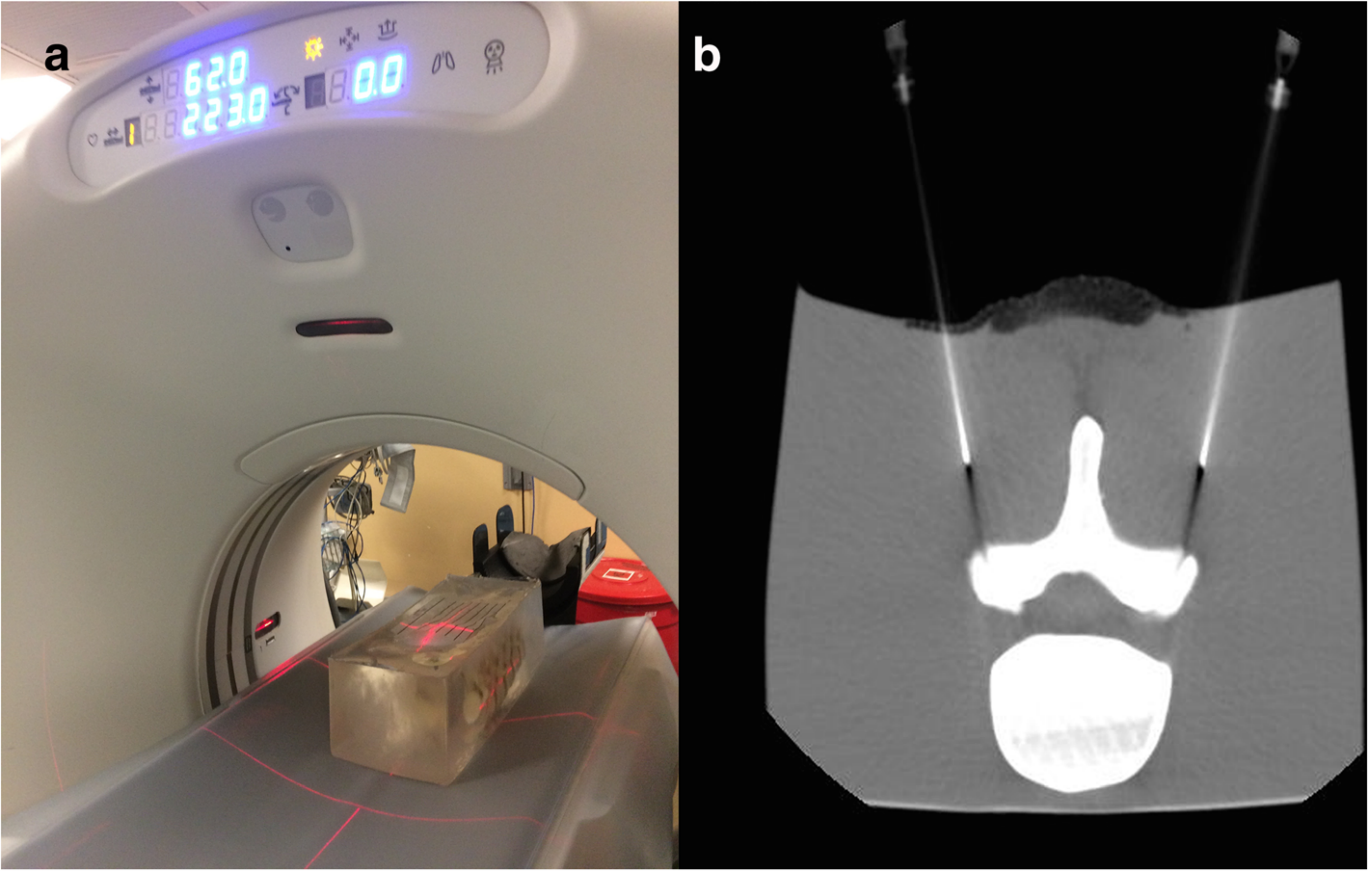
**a** Phantom spine model embedded in gelatin, with overlying grid, positioned within the CT scanner in preparation for training procedure. **b** CT image obtained during training session of two spinal needles in proper trajectory to achieve final position within bilateral facet joints

The phantom model demonstrates realistic spondylosis (Fig. 2) and is visible by both fluoroscopy (Fig. 3) and CT (Fig. 4). When imaged by CT, the osseous structures in the phantom model were confirmed to demonstrate bone density Hounsfield units (Fig. 4). The intervertebral discs and nerve roots demonstrated distinguishable densities under CT compared to bone (Figs. 5 and 6). The model was printed to us by Mediprint, Inc. free of charge, but cost approximately $5400 to manufacture, accounting for digital engineering, materials and assembly. The process of creating the model took approximately one month, accounting for selection of materials, printing and revisions to the density of materials.

### Knowledge-based examination

There was no statistical difference between the baseline knowledge of the Control group (1.8 ± 0.75) and Training group (2.3 ± 0.95), as evidenced by their performance on the pre-test (*p* < 0.37). Additionally, there was no statistical difference between knowledge of the Control group (3 ± 0) and Training group (2.7 ± 0.5) on the post-test after training (*p* < 0.18). The Control group performed significantly better on the post-test than the pre-test (*p* < 0.004, Table 1). The Training group, however, did not perform significantly better on the post-test than the pre-test (*p* < 0.31, Table 1).

**Table 1:** Differences in performance between the pre-training and post-training on the written exam, self-expressed interest in diagnostic radiology, confidence in ability to perform CT-guided spine procedures, and number of needle readjustments required to achieve target location, as compared between the Control group and Training group

	Control Group			Training Group		
	Pre-training	Post-training		Pre-training	Post-training	
Variable	Mean ± SD	Mean ± SD	p	Mean ± SD	Mean ± SD	p
Exam Score	1.8 ± 0.75	3 ± 0	0.004	2.3 ± 0.95	2.7 ± 0.5	0.31
Confidence in Ability	1.83 ± 2.0	5.8 ± 1.6	0.004	1 ± 0	6.1 ± 1.1	0.00001
Needle Adjustments	7.2 ± 1.5	x	x	8.0 ± 1.3	5.4 ± 1.5	0.005

### Self-reported confidence in ability to perform a CT-guided spine procedure

There was no statistically significant difference between the Control group (1.83 ± 2.0) and the Training group’s (2.3 ± 0.95) pre-training self-reported confidence in ability to perform CT-guided spine procedure (*p* < 0.30). Additionally, there was no statistically significant difference between the Control group (5.8 ± 1.6) and Training group’s (6.1 ± 1.1) post-training self-reported confidence in ability to perform CT-guided spine procedure (*p* < 0.69). For the Control group, there was a statistically significant increase in pre-training and post-training confidence in ability (*p* < 0.004, Table 1). For the Training group as well, there was a statistically significant increase in confidence in ability pre- and post-training (*p* < 0.00001, Table 1).

### Performance metrics

During the initial training session using the spine phantom (the only training session for the Control group), there was no significant difference between the two groups with regards to the number of needle readjustments required to achieve optimal positioning in the facet joints (*p* < 0.34). For the Training group that received an additional training session, there was a statistically significant reduction in the number of needle adjustments required to achieve proper position within the facet joints in the final session compared to the initial session (*p* < 0.005, Table 1). The radiation dose required to perform the facet injections was not directly tracked for each participant, however, using our standard low dose 10 mA technique, the average DLP to scan one vertebral body level in a standard size adult is approximately 1.5 mGy-cm, and thus, an average reduction of 3 needle readjustments would result in an overall reduction in 4.5 mGy-cm of dose per level injected.

## Discussion

Minimally-invasive CT-guided spine procedures are commonly performed in radiology departments to provide targeted, individualized diagnostic testing and pain management to patients. The ability for novice trainees to learn and practice these procedures on a phantom model would limit intra-procedural risk and radiation dose to patients. To the best of our knowledge, no study to date has evaluated the efficacy of using a realistic 3D printed lumbar spine phantom to train novice practitioners on the performance of CT-guided procedures. The results of our study indicate that a training curriculum using such a phantom model would increase confidence and proficiency in performing CT-guided spine procedures.

The novice trainees recruited into the study had no experience with CT-guided spine procedures, and, as expected, there was no significant difference between the pre-test knowledge of the Control and Training groups. The Control group, which only received one training session, took the post-test on the same day as the pre-test, shortly after receiving the didactic lecture, and thus performed slightly better on the post-test as compared to the Training group, which took their written post-test on a return session separated by approximately 2 weeks. Subsequent to the training sessions, both the Training and Control groups reported a statistically significant increase in their confidence in performing CT-guided spine procedures.

Regarding the technical aspect of the training, after only one additional training session, the Training group demonstrated a significant reduction in the number of needle adjustments required to achieve final needle position within the facet joint, suggesting that training increases mechanical proficiency. Fewer and more precise adjustments translate to better patient safety when trainees perform these procedures in the real clinical setting. Since a new CT scan is acquired each time the needle is readjusted, fewer needle readjustments also correlates directly to lower radiation dose.

Several issues must be considered when deciding the proper method by which novice trainees can gain procedural experience. Many procedures can be learned through real-time performance on patients in clinical practice, under the supervision of an experienced practitioner. For more technically challenging and high-risk procedures, however, this training method raises the ethical issue of patient risk. Such procedures can be practiced on cadavers, but cadavers may be expensive and difficult to access. Animal models are also expensive and difficult to access, and are associated with issues related to animal-borne diseases and comparative anatomy. Prior studies have proposed the use of commercially available spine models for the practice of spine interventions [4], but these models lack realistic degenerative characteristics that make real-life spine procedures challenging. Thus, we concluded that a 3D printed phantom of a realistic degenerative lumbar spine embedded in gelatin is the best training tool, as it mimics the complexities of a real patient spine and demonstrates both life-like tactile feedback and realistic radiographic density.

Several prior studies on phantom-based training have used various performance metrics to assess technical proficiency. Some studies using phantom models to practice ultrasound-guided spine procedures have used the overall time required for task completion [11], number of needle re-entries [11], number of needle passes [12], ergonomics [5], localization of the lesion [5], appropriate needle insertion [5], visualization of the needle throughout the procedure [5], and proper placement of the needle on the target lesion [5]. Other studies on minimally-invasive surgery have used time to completion of surgery, an anatomic check-list, and a safety-check list [10]. In our study, we use the number of needle readjustments required to achieve final needle position as the performance metric because it serves as a corollary of several factors: proper skin entry position, number of CT acquisitions required, overall procedural time, and radiation dose.

Similar to our experience with CT, one prior study using ultrasound has found that a similar spine model embedded in gelatin demonstrates realistic tissue-like texture for the practice of sonographically-guided spinal and epidural steroid injections [3]. Kwon et al. [5] used a spine phantom embedded in gelatin to teach ultrasound-guided facet injections and medial branch blocks to novice operators and achieved results similar to our study. The training group demonstrated increased confidence as well as increased proficiency at these procedures, as evidenced through a faster overall procedure time.

Our study has several limitations. The study may have been underpowered to detect some statistical differences. Due to limitations in student and scanner availability, the Training group only received one additional training session compared to the Control group. In the absence of a larger sample size, more prolonged training may have accentuated the findings of the current study, supporting the benefits of training with a phantom model. With sufficient training, a previously inexperienced operator may be able to achieve the same level of proficiency as an experienced operator, who may be able to perform a facet injection after only 2–3 needle manipulations. Additionally, the study focused on the performance of CT-guided facet blocks, which may not be generalizable to other procedures, such as CT-guided transforaminal nerve blocks, interlaminar epidural steroid injections, or other more complex procedures. Finally, as an inanimate phantom was used in the training sessions, the results of reported self-confidence may reflect an inflated false confidence, since secondary issues associated real patients, such as movement, pain, and positioning, were not accounted for.

For future investigation and training, additional spine training models can be made based off of differing patient anatomy, including cervical spine models. As no two patients have similar anatomy, introducing a different model during subsequent training sessions can test the trainee’s ability to synthesize knowledge and problem solve in real-time. Additionally, involving radiology residents and fellows in such training will allow trainees at various stages of knowledge and technical acumen to improve their skills at spine procedures.

## Conclusion

The results of this study indicate that training with a realistic 3D printed lumbosacral spine model helps novice trainees to acquire the technical proficiency and confidence to perform CT-guided minimally-invasive spine procedures.

## Abbreviations

3D: three dimensional
CT: computed tomography
STL: stereolithography
